# Optimising dynamic mechanical analysis experiments on soft rubbers for use in time temperature superposition

**DOI:** 10.1016/j.mex.2022.101831

**Published:** 2022-08-27

**Authors:** A.R. Trivedi, N. Hawkins, C.R. Siviour

**Affiliations:** University of Oxford, Engineering Science, Oxford, Oxfordshire, United Kingdom

**Keywords:** Viscoelasticity, High strain rate, Thermomechanical characterisation, Rate and temperature dependence

## Abstract

Rubbers are ubiquitous in engineering applications where they are often subjected to loading leading to high strain rate deformation. The strong rate and temperature dependence of rubbers and their composites motivates research into understanding their mechanical response under a wide range of conditions. However, experimental characterisation of the rate-temperature dependence of soft rubbers is challenging. In this methods paper, an improved methodology is proposed for conducting Dynamic Mechanical Analysis (DMA) experiments on rubbers. The higher quality data produced can be used in time-temperature superposition (TTS) applications to derive a more accurate definition of the rubber's rate-temperature dependence. Overall, the improvements obtained can be summarised as follows:

• Overall, the proposed methodology can be summarised with the following improvements:
• Reducing clamping artefacts due to volume expansion
• Ensuring high quality temperature stability
• Improving the contact area between the specimen and the clamps

## GraphicalAbstract_DMA

**Figure:**
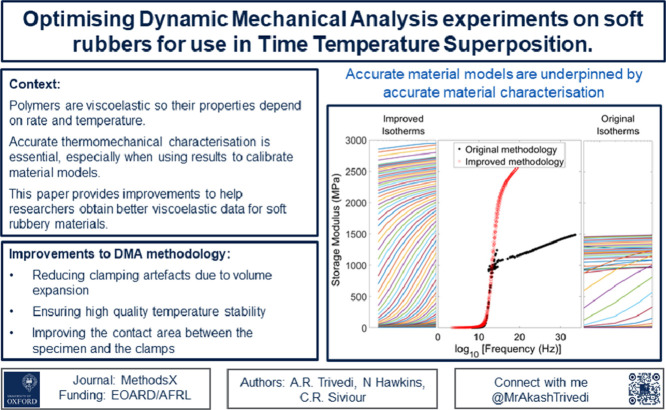

Specifications table

**Table:** 

Subject Area:	Engineering
More specific subject area:	Experimental mechanics, dynamic behaviour of materials, thermo-mechanics, polymer rheology
Method name:	Enhanced DMA for soft rubbers
Name and reference of original method:	BS ISO 18437-6:2017 [1], ISO 6721-11:2019 [2], Previous studies [3,4]
Resource availability:	Suitable DMA apparatus (TA Instruments Q800 in this study)

*Method details See attached PDF file accompanying this document.

## Declaration of interests

N/A
